# Diagnostic Value of the Blood Neutrophil-to-Lymphocyte Ratio and Monocyte-to-Lymphocyte Ratio in Tibia Fracture-Related Infection

**DOI:** 10.1155/2022/6119583

**Published:** 2022-06-02

**Authors:** Peisheng Chen, Yinhuan Liu, Xiaofeng Lin, Susu Tang, Tongtong Wang, Ke Zheng, Dongze Lin, Chaohui Lin, Bin Yu, Bin Chen, Fengfei Lin

**Affiliations:** ^1^Department of Orthopaedics, Fuzhou Second Hospital, The Third Clinical Medical College, Fujian Medical University, Fuzhou 350007, China; ^2^Department of Orthopaedics, Fuzhou Second Hospital of Xiamen University, School of Medicine, Xiamen University, Fuzhou 350007, China; ^3^Fujian Provincial Clinical Medical Research Center for First Aid and Rehabilitation in Orthopaedic Trauma, Fuzhou Trauma Medical Center, Fuzhou 350007, China; ^4^Department of Laboratory Medicine, Fuzhou Second Hospital of Xiamen University, School of Medicine, Xiamen University, Fuzhou 350007, China; ^5^Department of Endocrinology, Fuzhou Second Hospital, Fuzhou 350007, China; ^6^Department of Orthopaedics, Fuzhou Second Hospital, The Second Clinical Medical College, Fujian University of Traditional Chinese Medicine, Fuzhou 350007, China; ^7^Division of Orthopaedics & Traumatology, Department of Orthopaedics, Nanfang Hospital, Southern Medical University, Guangzhou 510515, China

## Abstract

**Objective:**

The diagnostic value of neutrophil-to-lymphocyte ratio (NLR), monocyte-to-lymphocyte ratio (MLR), and platelet-to-lymphocyte ratio (PLR) in predicting fracture-related infection (FRI) in tibia fracture patients remains to be explored.

**Methods:**

A retrospective controlled study was carried out with 170 tibia FRI patients and 162 control subjects. The following information was evaluated at admission: age, gender, clinical features, number of white blood cells (WBCs), neutrophils, lymphocytes, monocytes, red blood cells (RBCs), platelets, level of hemoglobin, C-reactive protein (CRP), and erythrocyte sedimentation rate (ESR), as well as NLR, MLR, and PLR.

**Results:**

The number of lymphocytes, RBCs, and platelets in the FRI group was higher than those in the control group, while the number of neutrophils and ESR level was lower (*P* < 0.05). The level of NLR and MLR was significantly lower in patients with tibia FRI than in control subjects (*P* < 0.05). Both indicators were positively correlated with WBCs, CRP level, and ESR level (*P* < 0.001). The results of logistic regression analysis showed that five variables including NLR, MLR, platelets, fracture pattern (closed or open fracture), and site pattern (single or multiple site) were used to construct the FRI risk predictor. The ROC curve analysis result showed that FRI risk predictor yielded the highest AUC, with a sensitivity of 91.2% and a specificity of 90.1%, and made the distinction efficiently between tibia FRI patients and non-FRI patients.

**Conclusion:**

NLR and MLR were decreased in tibia FRI patients compared to non-FRI patients. Both indicators had a positive correlation with WBCs, CRP level, and ESR level. FRI risk predictor constructed based on five variables including NLR and MLR had a high diagnostic value for tibia FRI.

## 1. Introduction

Fracture-related infection (FRI) is a serious and complex consequence related to bone injury [1], which brings an enormous financial burden and even great psychological pressure to patients and their families. The tibia is the most commonly affected bone (64%) [2]. Tibia fractures have a high risk of developing FRI ranging from approximately 26% to 61% in China [3–7], especially those with high-energy trauma, open fracture, and multiple sites injury.

Due to the diversity of clinical manifestations of FRI, there is a lack of unique and typical clinical symptoms, especially in the early postoperative period [8]. It is worth noting that the uncertainty of the early diagnosis in these patients may hinder the early and appropriate treatment, often leading to the chronic development of FRI. Therefore, in addition to routine medical history taking and physical examination, the detection of inflammatory markers, including white blood cells (WBCs), C-reactive protein (CRP), and erythrocyte sedimentation rate (ESR), is also indispensable to provide clues for the early diagnosis of FRI. However, a latest meta-analysis shows that the diagnostic value of these 3 markers is limited [9]. The FRI Consensus Group recommends that abnormal elevations of these inflammatory markers be used as a suggestive criterion for the diagnosis of FRI [1]. This indicates that there is an urgent need for novel inflammatory markers for the diagnosis of FRI at an earlier disease stage.

Many cells in the blood, such as neutrophils, lymphocytes, and monocytes, play a vital role in the innate immune defense process against pathogen invasion. Recently, growing evidence has indicated that neutrophil-to-lymphocyte ratio (NLR), monocyte-to-lymphocyte ratio (MLR), and platelet-to-lymphocyte ratio (PLR) can be used as effective and inexpensive makers in the diagnosis of many inflammatory and immune diseases including fever caused by bacterial infection [10], incident TB infection [11], community-acquired pneumonia [12], critically ill patients with secondary sepsis and/or trauma [13], and peripheral arterial disease, peripheral neuropathy, osteomyelitis, and need for amputation in diabetic foot infection [14]. Similarly, the expression levels of NLR, PLR, and MPV in autoimmune diseases such as systemic lupus erythematosus, rheumatoid arthritis, and ankylosing spondylitis and their relationship with disease severity and treatment prognosis have also been extensive investigations [15–20].

Bone fractures are associated with inflammation [21]. Systemically, changes in NLR, PLR, and MLR represent primary responses to early inflammation and infection. When compared with healthy controls, patients with humeral, femoral, and tibial diaphyseal fractures had significantly higher neutrophil numbers, NLR, and lower thrombocyte numbers [22]. Elevated NLR appeared to predict more severe tibial plateau fractures [23]. In patients with intertrochanteric fractures older than 90 years treated with proximal femoral nailing, WBC, NLR, and PLR values were also elevated but were not significantly correlated with survival [24]. Early postoperative NLR may help to recognize postoperative delirium in elderly patients undergoing surgery for lower limb fracture under nongeneral anaesthesia [25]. On the other hand, the predictive value of these markers for the prognosis of hand osteomyelitis, surgical site infection after spinal instrumentation surgery, and periprosthetic joint infection following total hip arthroplasty also needs to be further studied [26–28].

Therefore, it can be hypothesized that these markers would be associated with inflammation related to tibia fracture. In this study, we aim to investigate the diagnostic value of NLR, MLR, and PLR in tibia FRI patients.

## 2. Materials and Methods

### 2.1. Patient Characteristics

This study, designed as a retrospective controlled analysis, reviewed the electronic medical records of patients hospitalized for tibia fractures in two tertiary healthcare centers, Fuzhou Second Hospital of Xiamen University and Southern Medical University Nanfang Hospital, from January 1, 2014, to December 31, 2018. Participants of the tibia FRI group were patients who fulfilled any of the following four primary diagnostic criteria for FRI referred to the international consensus by the Association for the Study of Internal Fixation (AO/ASIF) [1]: (1) fistula, sinus, or wound breakdown (with communication to the bone or the implant); (2) purulent drainage from the wound or presence of pus during surgery; (3) phenotypically indistinguishable pathogens identified by culture from at least two separate deep tissue/implant (including sonication-fluid) specimens taken during an operative intervention. In case of tissue, multiple specimens (≥3) should be taken, each with clean instruments (not superficial or sinus tract swabs). In cases of joint effusion, arising in a joint adjacent to a fractured bone, fluid samples obtained by sterile puncture may be included as a single sample; (4) presence of microorganisms in deep tissue taken during an operative intervention, as confirmed by histopathological examination using specific staining techniques for bacteria or fungi. Patients with autoimmune diseases, malignancy, severe heart, lung, liver, and kidney diseases were excluded. The control group, as the non-FRI group, was consisted of tibia fracture patients who had been confirmed with fracture healed in the following three months. All procedures performed in this study involving human participants were in accordance with the Declaration of Helsinki. This study has been approved by the medical ethical committee office of Fuzhou Second Hospital of Xiamen University.

### 2.2. Clinical and Laboratory Assessments

The following patient information at admission was recorded: age, gender, clinical features, number of WBCs, neutrophils, lymphocytes, monocytes, red blood cells (RBCs), and platelets, as well as level of hemoglobin, CRP, and ESR. NLR, MLR, and PLR were calculated.

### 2.3. Statistical Analysis

All data analyses were performed with IBM SPSS Statistics for Windows, version 21.0 (IBM Corp., Armonk, NY, USA). Normally and skewed distributed continuous variables were expressed as mean ± standard deviation and median (Q1, Q3), respectively. Normally distributed data were analyzed as independent samples using the Student's *t*-test, and data with a skewed distribution were analyzed by Mann–Whitney *U* tests. Categorical variables were summarized as number (*n*) and percentage (%). Comparisons of categorical variables between two groups were assessed with Chi-squared tests. To test for associations, normally distributed continuous data were analyzed using the Pearson correlation, while other data using the Spearman correlation. The effects of different variables on FRI were further analyzed by multivariate binary logistic regression analysis. The receiver operating characteristic (ROC) curve analysis was performed to analyze the differential diagnostic value (such as sensitivity, specificity, and area under the curve (AUC)) of NLR, MLR, platelets, and FRI risk predictor for tibia FRI. A *P* value < 0.05 was considered statistically significant.

## 3. Results

### 3.1. Basic Characteristics of Tibia FRI Patients and Control Subjects

A total of 170 tibia FRI patients and 162 control subjects were included in the study.  Table 1 presents the main laboratory and clinical characteristics. There were significant differences between the two groups in terms of gender and open or closed fracture (*P* < 0.001). The number of lymphocytes, RBCs, and platelets in the FRI group was higher than those in the control group, while the number of neutrophils and ESR level was lower (*P* < 0.05). Positive rates of inflammation markers showed that ESR was the highest (65.66%, 218/332), followed by CRP (31.02%, 103/332) and WBCs (13.25%, 44/332), respectively. Statistical analysis for positive rates of WBCs and ESR between the two groups showed significant differences (*P* < 0.05) (Table 1).

### 3.2. NLR and MLR Were Decreased in Tibia FRI Patients

**Figure 1:**
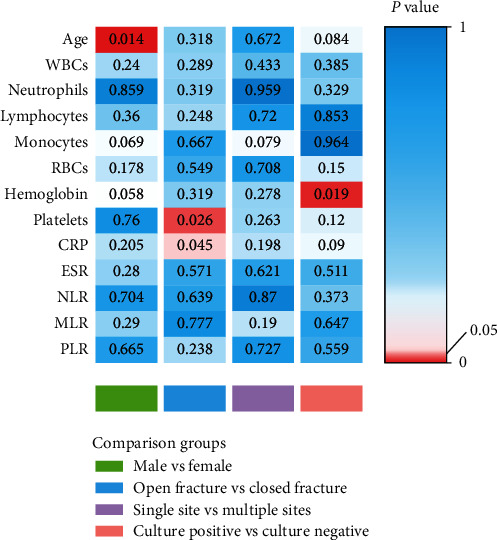
Differences in age and laboratory findings between different types of tibia FRI patients. The data and the corresponding color indicated the *P* value. WBCs: white blood cells; RBCs: red blood cells; CRP: C-reactive protein; ESR: erythrocyte sedimentation rate; NLR: neutrophil-to-lymphocyte ratio; MLR: monocyte-to-lymphocyte ratio; PLR: platelet-to-lymphocyte ratio.

The level of NLR and MLR was significantly lower in patients with tibia FRI than in control subjects (*P* < 0.01) (Table 1). However, there was no statistically significant difference between different types of tibia FRI patients in terms of NLR, MLR, or PLR (Figure 1).

### 3.3. NLR, MLR, and PLR Were Associated with CRP Level and ESR Level

**Figure 2:**
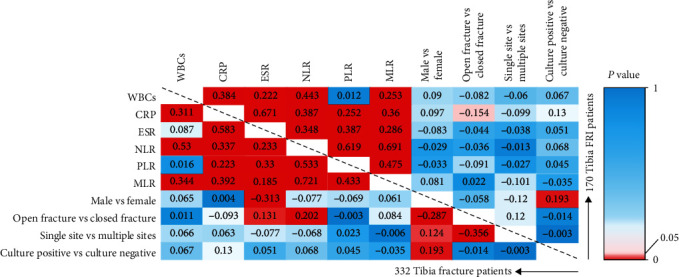
Analysis of the correlation between variables. The data corresponded to the correlation coefficient, and the corresponding color indicated the *P* value. WBCs: white blood cells; CRP: C-reactive protein; ESR: erythrocyte sedimentation rate; NLR: neutrophil-to-lymphocyte ratio; MLR: monocyte-to-lymphocyte ratio; PLR: platelet-to-lymphocyte ratio.

In tibia FRI patients, NLR and MLR were all positively correlated with WBCs (*r* = 0.443 and *P* < 0.001 and *r* = 0.253 and *P* < 0.001, respectively), CRP level (*r* = 0.387 and *P* < 0.001 and *r* = 0.360 and *P* < 0.001, respectively), and ESR level (*r* = 0.348 and *P* < 0.001 and *r* = 0.286 and *P* < 0.001, respectively). However, PLR was only positively correlated with CRP level (*r* = 0.252, *P* < 0.001) and ESR level (*r* = 0.387, *P* < 0.001). These were consistent with the trends observed in all tibia fracture patients (Figure 2).

### 3.4. FRI Risk Predictor Constructed by Logistic Regression Analysis

Association of demographic features and laboratory tests of FRI patients was further analyzed by multivariate binary logistic regression analysis. Finally, five variables including NLR, MLR, platelets, fracture pattern (closed or open fracture), and site pattern (single or multiple site) were used to construct the FRI risk predictor (Hosmer-Lemeshow *χ*^2^ = 10.224, *P* = 0.250) (Table 2). According to the results of logistic regression analysis, the risk score of FRI in patients with tibia fractures was logit (*P*) = −3.691 − 1.082∗NLR + 5.342∗MLR + 0.008∗Platelets + 3.661∗Fracture pattern (closed fracture = 0; open fracture = 1) + 3.034∗Site pattern (single site = 0; multiple site = 1).

### 3.5. FRI Risk Predictor Has a High Diagnostic Value for Tibia FRI by ROC Curve Analysis

**Figure 3:**
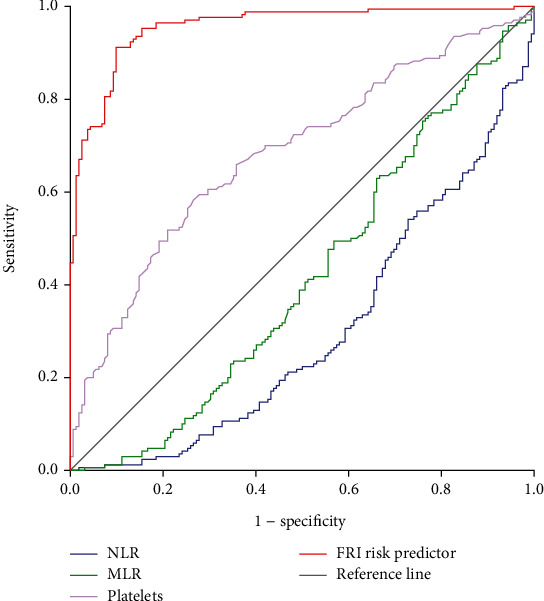
ROC curve analysis of the diagnostic value of NLR, MLR, platelets, and FRI risk predictor for tibia FRI. ROC: receiver operating characteristic; NLR: neutrophil-to-lymphocyte ratio; MLR: monocyte-to-lymphocyte ratio; FRI risk predictor: *e*^logit(*P*)^/(1 + *e*^logit(*P*)^).

The results of ROC curve analysis showed that the AUC of NLR, MLR, platelets, and FRI risk predictor for tibia FRI were 0.299 (95% confidence interval (CI): 0.244-0.355), 0.417 (95% CI: 0.355-0.478), 0.684 (95% CI: 0.627-0.741), and 0.955 (95% CI: 0.935-0.976), respectively (*P* < 0.05) (Figure 3 and Table 3). FRI risk predictor yielded the highest AUC, with a sensitivity of 91.2% and a specificity of 90.1%.

## 4. Discussion

Although the current diagnosis and treatment concepts have been advanced, FRI continues to be a thorny issue that plagues the majority of orthopedic surgeons [29]. The importance of early diagnosis is self-evident, which has prompted the research of novel inflammatory markers for the diagnosis of FRI at an earlier disease stage, and has gradually attracted more and more attention. This retrospective controlled study explored the promising diagnostic value of NLR, MLR, and PLR for FRI in patients with tibia fractures. In this study, we found that NLR and MLR were decreased and positively correlated with the WBCs, CRP level, and ESR level in tibia FRI patients. The results of logistic regression analysis and ROC curve analysis showed that the FRI risk predictor constructed based on five variables including NLR and MLR had a high diagnostic value for FRI in tibia fracture patients.

During infection in patients with tibia FRI, the immune system produces an inflammatory response to neutralize or kill pathogens, maintaining a trade-off between clearing pathogens and limiting damage to host tissues, and eventually leading to recovery or tissue healing [30]. Changes in the content of immune cells, including neutrophils, lymphocytes, and monocytes, easily obtained from complete blood counts, can reflect the balance between the immune status of the body and the invasion status of pathogens to a certain extent.

Neutrophils are the most abundant circulating leukocyte type in humans and play important roles in the first line of defense against foreign pathogens [31]. The signal pathways related to neutrophil activation and the formation of neutrophil extracellular traps may affect the occurrence and development of FRI [32]. In addition, lymphocytes act as a key cell type involved in directing and propagating the adaptive immune response, providing a broader and more finely tuned repertoire of recognition for both self- and nonself-antigens [33]. Macrophage-derived chemokines induce macrophages and osteoclast precursor cells to polarize and migrate to inflammatory tissues to participate in the pathogenesis of osteomyelitis, whereas inflammatory cytokines promote osteoclast differentiation and inhibit osteoblast formation [34]. The activation of platelets participated in the pathogenesis of thrombosis in association with *Staphylococcus aureus* osteomyelitis [35]. Thus, changes in the numbers of immune cells have a great impact on the direction of the body's inflammatory response in patients with tibia fractures.

Previous studies had addressed the role of NLR, MLR, and PLR in infections and characterized the clinical prediction potential for bacterial infection and nonspecific infection [10–14]. In terms of research related to bone infections, these immune-related biomarkers, such as WBCs, CRP, ESR, NLR, MLR, and PLR, are increasingly being evaluated for their abilities of fast and early diagnosis of osteomyelitis and to predict complications [26–28, 36–38]. In diabetic foot infection patients, PLR was significantly higher in osteomyelitis, and the cut-off value PLR > 187.3 had a sensitivity of 67.9% and a specificity of 59.1% in predicting osteomyelitis [14]. Moreover, NLR, PLR, and MLR were predictive of the need for amputation in diabetic foot infection. Patients who required amputation had higher NLR and PLR compared with those who did not. NLR was significant in determining the level of their amputation, either minor or major.

However, the diagnostic and prognostic values of the inflammatory parameters of NLR, PLR, and MLR for FRI have not been revealed yet. van den Kieboom et al. reported in their systematic review that the diagnostic accuracy of the serum inflammatory markers WBCs, CRP, and ESR was insufficient to diagnose or exclude late FRI, with a sensitivity of 51.7%, 77.0%, and 45.1%, respectively, and a specificity of 67.1%, 67.9%, and 79.3%, respectively [9]. In the current study, the number of lymphocytes, RBCs, and platelets was higher in patients with FRI than in patients with only tibia fractures, whereas the number of neutrophils, ESR level, NLR, and MLR was lower. Interestingly, subgroup analysis of tibia FRI patients according to gender (male or female), fracture pattern (closed or open fracture), site pattern (single or multiple site), and bacterial culture results (positive or negative) found that most of these inflammatory markers did not have statistical differences. In addition, there was a clear positive correlation between these inflammatory markers. This suggested that the potential application value of each marker cannot be analyzed individually but should be understood comprehensively in the research of inflammation markers associated with FRI. We further analyzed the relationship between the demographic characteristics of FRI patients and these detection indicators through logistic regression analysis and made a risk assessment of FRI in these patients with tibia fractures. The result showed that FRI risk predictor occupied the highest AUC, with a sensitivity of 91.2% and a specificity of 90.1%, and acted as an efficient indicator to make the distinction between tibia FRI patients and non-FRI patients. Therefore, NLR and MLR may be more useful diagnostic tools than other more commonly used diagnostic blood tests to early identify patients with tibia FRI.

There were some limitations in our study. First, this was a retrospective controlled study involving only two centers, so more patients from multicenter should be evaluated in future research. Second, we did not investigate the pathogenesis of decreased NLR and MLR. Therefore, longitudinal and molecular biology studies are needed to explore the potential mechanism. Third, we only used the separate data at admission of the FRI group and the control group for comparison. There was a lack of data on inflammatory markers before infection in FRI patients.

## 5. Conclusions

In this study, NLR and MLR were decreased in tibia FRI patients compared to non-FRI patients. Both indicators had a positive correlation with WBCs, CRP level, and ESR level. The NLR and MLR could be potential diagnostic factors for tibia FRI. FRI risk predictor constructed based on five variables including NLR, MLR, platelets, fracture pattern (closed or open fracture), and site pattern (single or multiple site) had a high diagnostic value for tibia FRI. However, more research will be indispensable for the further clinical application of these indicators.

## Figures and Tables

**Table 1:** Demographic features and laboratory findings of the participants.

	Control (*n* = 162)	FRI (*n* = 170)	*P* value
Age (years)	39 (28, 50)	42 (28.50, 49)	0.976
Gender (male/female)	95/67	145/25	<0.001
WBCs (×10^9^/L)	7.50 (6.10, 9.13)	7.13 (5.96, 8.34)	0.079
Neutrophils (×10^9^/L)	4.83 (3.86, 6.93)	4.16 (3.17, 5.39)	<0.001
Lymphocytes (×10^9^/L)	1.73 (1.46, 2.07)	2.05 (1.71, 2.50)	<0.001
Monocytes (×10^9^/L)	0.47 (0.36, 0.64)	0.51 (0.40, 0.63)	0.174
RBCs (×10^12^/L)	4.41 (4.09, 4.82)	4.72 (4.22, 5.05)	0.002
Hemoglobin (g/L)	133 (121, 145)	132.50 (114.75, 144.25)	0.251
Platelets (×10^9^/L)	227 (188.75, 267.25)	278 (218.75, 353.25)	<0.001
CRP (mg/L)	2.90 (1.58, 8.82)	4.49 (1.72, 10.04)	0.244
ESR (mm/h)	25 (15, 41)	20 (8, 34.25)	0.005
NLR	2.73 (1.86, 4.35)	1.97 (1.40, 2.55)	<0.001
MLR	0.26 (0.19, 0.39)	0.24 (0.19, 0.32)	0.009
PLR	132.70 (105.49, 174.56)	133.31 (99.29, 174.05)	0.694
Positive rates of inflammation markers (events, %)			
WBCs	30, 18.52%	14, 8.24%	0.006
CRP	50, 30.86%	53, 31.18%	0.951
ESR	121, 74.69%	97, 57.06%	0.001
Open fracture vs. closed fracture	15/147	126/44	<0.001
Single site vs. multiple sites	118/44	137/33	0.094
Positive rate of culture (events, %)		87, 51.18%	
Monomicrobial infection vs. polymicrobial infection		67/20	

FRI: fracture-related infection; WBCs: white blood cells; RBCs: red blood cells; NLR: neutrophil-to-lymphocyte ratio; MLR: monocyte-to-lymphocyte ratio; PLR: platelet-to-lymphocyte ratio; CRP: C-reactive protein; ESR: erythrocyte sedimentation rate.

**Table 2:** Variables associated with FRI by multivariable binary logistic regression analysis.

Variable	Regression coefficient	SEM	Wald *χ*^2^ value	OR (95% CI)	*P* value
NLR	-1.082	0.229	22.285	0.339 (0.216-0.531)	< 0.001
MLR	5.342	2.198	5.909	208.929 (2.815-15507.615)	0.015
Platelets	0.008	0.002	13.191	1.008 (1.004-1.012)	< 0.001
Fracture pattern (closed or open fracture)	3.661	0.477	58.950	38.915 (15.283-99.091)	< 0.001
Site pattern (single or multiple site)	3.034	0.437	48.300	20.783 (8.833-48.902)	< 0.001
Constant	-3.691	0.79	21.811	—	< 0.001

SEM: standard error of mean; OR: odds ratio; CI: confidence interval; NLR: neutrophil-to-lymphocyte ratio; MLR: monocyte-to-lymphocyte ratio.

**Table 3:** The AUC of NLR, MLR, platelets, and FRI risk predictor for the diagnosis of tibia FRI.

Variable	AUC (95% CI)	SEM	*P* value	Youden index	Cut-off value	Sensitivity	Specificity
NLR	0.299 (0.244-0.355)	0.028	< 0.001	0	—	—	—
MLR	0.417 (0.355-0.478)	0.031	0.009	0.015	0.132	94.7%	6.8%
Platelets	0.684 (0.627-0.741)	0.029	< 0.001	0.317	262.500	58.2%	73.5%
FRI risk predictor	0.955 (0.935-0.976)	0.011	< 0.001	0.813	0.554	91.2%	90.1%

AUC: area under the curve; SEM: standard error of mean; NLR: neutrophil–lymphocyte ratio; MLR: monocyte–lymphocyte ratio; FRI risk predictor: *e*^logit(*P*)^/(1 + *e*^logit(*P*)^).

## Data Availability

The data used to support the findings of this study are available from the corresponding author and first author upon request.

## References

[1] Metsemakers W. J., Morgenstern M., McNally M. A. (2018). Fracture-related infection: a consensus on definition from an international expert group. *Injury*.

[2] Bezstarosti H., van Lieshout E. M. M., Voskamp L. W. (2019). Insights into treatment and outcome of fracture-related infection: a systematic literature review. *Archives of Orthopaedic and Trauma Surgery*.

[3] Wang B., Xiao X., Zhang J., Han W., Hersi S. A., Tang X. (2021). Epidemiology and microbiology of fracture-related infection: a multicenter study in Northeast China. *Journal of Orthopaedic Surgery and Research*.

[4] Ma X., Han S., Ma J. (2018). Epidemiology, microbiology and therapeutic consequences of chronic osteomyelitis in northern China: a retrospective analysis of 255 patients. *Scientific Reports*.

[5] Wang X., Yu S., Sun D. (2017). Current data on extremities chronic osteomyelitis in southwest China: epidemiology, microbiology and therapeutic consequences. *Scientific Reports*.

[6] Peng J., Ren Y., He W. (2017). Epidemiological, clinical and microbiological characteristics of patients with post-traumatic osteomyelitis of limb fractures in Southwest China: a hospital-based study. *J Bone Jt Infect*.

[7] Jiang N., Ma Y. F., Jiang Y. (2015). Clinical characteristics and treatment of extremity chronic osteomyelitis in southern China: a retrospective analysis of 394 consecutive patients. *Medicine (Baltimore)*.

[8] Govaert G., Kuehl R., Atkins B. L. (2020). Diagnosing fracture-related infection: current concepts and recommendations. *Journal of Orthopaedic Trauma*.

[9] van den Kieboom J., Bosch P., Plate J. D. J. (2018). Diagnostic accuracy of serum inflammatory markers in late fracture-related infection. *Bone Joint J*.

[10] Naess A., Nilssen S. S., Mo R., Eide G. E., Sjursen H. (2017). Role of neutrophil to lymphocyte and monocyte to lymphocyte ratios in the diagnosis of bacterial infection in patients with fever. *Infection*.

[11] Rees C. A., Pineros D. B., Amour M. (2020). The potential of CBC-derived ratios (monocyte-to-lymphocyte, neutrophil-to-lymphocyte, and platelet-to-lymphocyte) to predict or diagnose incident TB infection in Tanzanian adolescents. *BMC Infectious Diseases*.

[12] Huang Y., Liu A., Liang L. (2018). Diagnostic value of blood parameters for community-acquired pneumonia. *International Immunopharmacology*.

[13] Djordjevic D., Rondovic G., Surbatovic M. (2018). Neutrophil-to-lymphocyte ratio, monocyte-to-lymphocyte ratio, platelet-to-lymphocyte ratio, and mean platelet volume-to-platelet count ratio as biomarkers in critically ill and injured patients: which ratio to choose to predict outcome and nature of bacteremia. *Mediators of Inflammation*.

[14] Demirdal T., Sen P. (2018). The significance of neutrophil-lymphocyte ratio, platelet-lymphocyte ratio and lymphocyte-monocyte ratio in predicting peripheral arterial disease, peripheral neuropathy, osteomyelitis and amputation in diabetic foot infection. *Diabetes Research and Clinical Practice*.

[15] Qin B., Ma N., Tang Q. (2016). Neutrophil to lymphocyte ratio (NLR) and platelet to lymphocyte ratio (PLR) were useful markers in assessment of inflammatory response and disease activity in SLE patients. *Modern Rheumatology*.

[16] Wu Y., Chen Y., Yang X., Chen L., Yang Y. (2016). Neutrophil-to-lymphocyte ratio (NLR) and platelet-to-lymphocyte ratio (PLR) were associated with disease activity in patients with systemic lupus erythematosus. *International Immunopharmacology*.

[17] Sargin G., Senturk T., Yavasoglu I., Kose R. (2018). Relationship between neutrophil-lymphocyte, platelet-lymphocyte ratio and disease activity in rheumatoid arthritis treated with rituximab. *International Journal of Rheumatic Diseases*.

[18] Du J., Chen S., Shi J. (2017). The association between the lymphocyte-monocyte ratio and disease activity in rheumatoid arthritis. *Clinical Rheumatology*.

[19] Al-Osami M. H., Awadh N. I., Khalid K. B., Awadh A. I. (2020). Neutrophil/lymphocyte and platelet/lymphocyte ratios as potential markers of disease activity in patients with ankylosing spondylitis: a case-control study. *Advances in Rheumatology*.

[20] Liang T., Chen J., Xu G. (2021). Platelet-to-lymphocyte ratio as an independent factor was associated with the severity of ankylosing spondylitis. *Frontiers in Immunology*.

[21] Loi F., Córdova L. A., Pajarinen J., Lin T. H., Yao Z., Goodman S. B. (2016). Inflammation, fracture and bone repair. *Bone*.

[22] Alexandru L., Haragus H., Deleanu B., Timar B., Poenaru D. V., Vlad D. C. (2019). Haematology panel biomarkers for humeral, femoral, and tibial diaphyseal fractures. *International Orthopaedics*.

[23] Wang Z., Tian S., Zhao K. (2020). Neutrophil to lymphocyte ratio and fracture severity in young and middle-aged patients with tibial plateau fractures. *International Orthopaedics*.

[24] Ergin O. N., Bayram S., Anarat F. B., Yağcı T. F., Balcı H. İ. (2020). Prognostic factors affecting survival of patients with intertrochanteric femoral fractures over 90 years treated with proximal femoral nailing. *European Journal of Trauma and Emergency Surgery*.

[25] Li X., Wang G., He Y., Wang Z., Zhang M. (2022). White-cell derived inflammatory biomarkers in prediction of postoperative delirium in elderly patients undergoing surgery for lower limb fracture under non-general anaesthesia. *Clinical Interventions in Aging*.

[26] Wyman M., Dargan D., Kazzazi D., Caddick J., Giblin V. (2022). Serum inflammatory markers and amputations in hand osteomyelitis: a retrospective review of 146 cases. *Hand (N Y)*.

[27] Kobayashi Y., Inose H., Ushio S. (2020). Body mass index and modified glasgow prognostic score are useful predictors of surgical site infection after spinal instrumentation surgery: a consecutive series. *Spine (Phila Pa 1976)*.

[28] Klemt C., Tirumala V., Smith E. J., Xiong L., Kwon Y. M. (2022). Complete blood platelet and lymphocyte ratios increase diagnostic accuracy of periprosthetic joint infection following total hip arthroplasty. *Archives of Orthopaedic and Trauma Surgery*.

[29] Chen P., Lin X., Chen B. (2021). The global state of research and trends in osteomyelitis from 2010 to 2019: a 10-year bibliometric analysis. *Ann Palliat Med*.

[30] Cicchese J. M., Evans S., Hult C. (2018). Dynamic balance of pro- and anti-inflammatory signals controls disease and limits pathology. *Immunological Reviews*.

[31] Nauseef W. M., Borregaard N. (2014). Neutrophils at work. *Nature Immunology*.

[32] Chen P., Yao Z., Deng G. (2018). Differentially expressed genes in osteomyelitis induced by Staphylococcus aureus infection. *Frontiers in Microbiology*.

[33] Zajonc D. M., Girardi E. (2015). Recognition of microbial glycolipids by natural killer T cells. *Frontiers in Immunology*.

[34] Josse J., Velard F., Gangloff S. C. (2015). Staphylococcus aureus vs. osteoblast: relationship and consequences in osteomyelitis. *Frontiers in Cellular and Infection Microbiology*.

[35] Niemann S., Bertling A., Brodde M. F. (2018). Panton-Valentine leukocidin associated with S. aureus osteomyelitis activates platelets via neutrophil secretion products. *Scientific Reports*.

[36] Lavery L. A., Ahn J., Ryan E. C. (2019). What are the optimal cutoff values for ESR and CRP to diagnose osteomyelitis in patients with diabetes-related foot infections?. *Clinical Orthopaedics and Related Research*.

[37] Michail M., Jude E., Liaskos C. (2013). The performance of serum inflammatory markers for the diagnosis and follow-up of patients with osteomyelitis. *The International Journal of Lower Extremity Wounds*.

[38] Xu J., Cheng F., Li Y., Zhang J., Feng S., Wang P. (2021). Erythrocyte sedimentation rate combined with the probe-to-bone test for fast and early diagnosis of diabetic foot osteomyelitis. *The International Journal of Lower Extremity Wounds*.

